# Exhaled and Systemic Biomarkers to Aid the Diagnosis of Bronchial Asthma in Elite Water Sports Athletes

**DOI:** 10.1249/MSS.0000000000003419

**Published:** 2024-03-06

**Authors:** BALÁzs CSOMA, NÓRA SYDÓ, GERGŐ SZŰcs, ÉVA SERES, TAMÁS ERDÉLYI, GÁBOR HORVÁTH, EMESE CSULAK, BÉLA MERKELY, VERONIKA MÜLLER

**Affiliations:** 1 Department of Pulmonology, Semmelweis University, Budapest, HUNGARY; 2 Heart and Vascular Centre, Semmelweis University, Budapest, HUNGARY

**Keywords:** BRONCHIAL ASTHMA, ELITE ATHLETES, EOSINOPHILIA, EXERCISE-INDUCED BRONCHOCONSTRICTION, F_ENO_, IGE, SKIN PRICK TEST

## Abstract

**Purpose:**

Our aim was to evaluate the accuracy of a combined airway inflammatory biomarker assessment in diagnosing asthma in elite water sports athletes.

**Methods:**

Members of the Hungarian Olympic and Junior Swim Team and elite athletes from other aquatic disciplines were assessed for asthma by objective lung function measurements, and blood eosinophil count (BEC), serum total immunoglobulin E (IgE), fractional exhaled nitric oxide (F_ENO_) measurements, and skin prick testing were performed. A scoring system from BEC, F_ENO_, serum IgE, and skin test positivity was constructed by dichotomizing the variables and assigning a score of 1 if the variable is elevated. These scores were summed to produce a final composite score ranging from 0 to 4.

**Results:**

A total of 48 participants were enrolled (age 21 ± 4 yr, 42% male), of which 22 were diagnosed with asthma. Serum total IgE and F_ENO_ levels were higher in asthmatic individuals (68 [27–176] vs 24 [1–43], *P* = 0.01; 20 [17–26] vs 15 [11–22], *P* = 0.02), and positive prick test was also more frequent (55% vs 8%, *P* < 0.01). Asthmatic participants had higher composite variable scores (2 [1–3] vs 1 [0–1], *P* = 0.02). Receiver operating characteristic analysis showed that total IgE, F_ENO_, and composite variable were suitable

for identifying asthmatic participants (area under the curve = 0.72, *P* = 0.01; 0.70, *P* = 0.02, and 0.69, *P* = 0.03). A composite score of >2 reached a specificity of 96.2%, a sensitivity of 36.4%, and a likelihood ratio of 9.5. Logistic regression model revealed a strong association between the composite variable and the asthma diagnosis (OR = 2.71, 95% confidence interval = 1.17–6.23, *P* = 0.02).

**Conclusions:**

Our data highlight the diagnostic value of combined assessment of Th2-type inflammation in elite water sports athletes. The proposed scoring system may be helpful in ruling in asthma in this population upon clinical suspicion.

Bronchial asthma is a common respiratory disease affecting more than 330 million people worldwide (1). Exercise-induced bronchoconstriction (EIB), which is characterized by acute onset of bronchospasm during or immediately after physical exertion, is a common feature of asthma (2). However, it can occur in nonasthmatic individuals as well (3,4), and its exact prevalence varies according to the criteria used for the diagnosis (2). Nonetheless, asthma and EIB are very common among elite athletes and may potentially limit the athletic performance (5–10). A recent systematic review and meta-analysis showed that the overall prevalence of lower airway dysfunction (a collective term for asthma, EIB, and airway hyperresponsiveness) is more than 20% among athletes; however, in elite athletes, it reaches almost 30%, whereas in aquatic disciplines, it is almost 40% (11).

Diagnostic tools for chronic inflammatory airway diseases involve basic (e.g., questionnaires, full blood count measurement, and spirometry with or without reversibility testing) and specific investigations (peak expiratory flow [PEF] variability measurement, direct (acting on airway smooth muscles, e.g., metacholine challenge) and indirect (acting on airway inflammatory cells, e.g., eucapnic voluntary hyperpnea) bronchial provocation tests, skin prick test, immunoglobulin E (IgE) measurement, etc.). The diagnosis of asthma is very challenging and requires specific considerations in elite aquatic athletes because they have often higher than normal lung function parameters, the symptoms are mostly present only during exertion and exercise in water, and the symptoms do not necessarily correlate with objective findings (5,12). Therefore, although clinical history and subjective symptoms are important in the diagnostic process, according to the recommendation of the International Olympic Committee, an objective test such as bronchial challenge should be performed (13). Nevertheless, the accessibility to such tests varies across regions and countries. However, certain exhaled and systemic biomarkers associated with asthma-specific and T helper (Th) 2-type inflammation can aid in the diagnosis (14). Blood eosinophil count (BEC), total IgE concentration, and skin prick test results are associated with airway allergy and Th2-type inflammation and may be used in phenotyping asthma (14). Elevated fractional exhaled nitric oxide (F_ENO_) is a marker of airway eosinophilic inflammation and a recommended diagnostic tool for bronchial asthma in the normal population (14,15). However, F_ENO_ levels in athletes may be different than of the general population, as highlighted by a recent study where 45% of the participants with EIB had values <25 ppb (16).

Consequently, we aimed to assess the clinical use of a complex biomarker assessment (cutaneous allergy testing, F_ENO_ measurement, BEC, and total IgE) for diagnosing asthma in a cohort of Hungarian elite water sports athletes.

## METHODS

### Participants

We included aquatic athletes who came for asthma screening as part of a sports medical assessment without symptoms, or came for the screening due to respiratory symptoms (such as cough, wheezing, breathlessness, and/or a sudden decrease in training performance) or asthma therapy optimization. In the frame of detailed sports medical screening, all members of the Hungarian Swim Team, participating in the Tokyo Olympics 2021, were screened for asthma (*N* = 41). We also performed the same screening for the National Junior Swim Team (*n* = 21) before the European Aquatic Championship 2022. Furthermore, we included symptomatic elite aquatic athletes from other disciplines (non-Olympic swimmers *n* = 8, water polo *n* = 8, Paralympic swimming *n* = 3).

**FIGURE 1:**
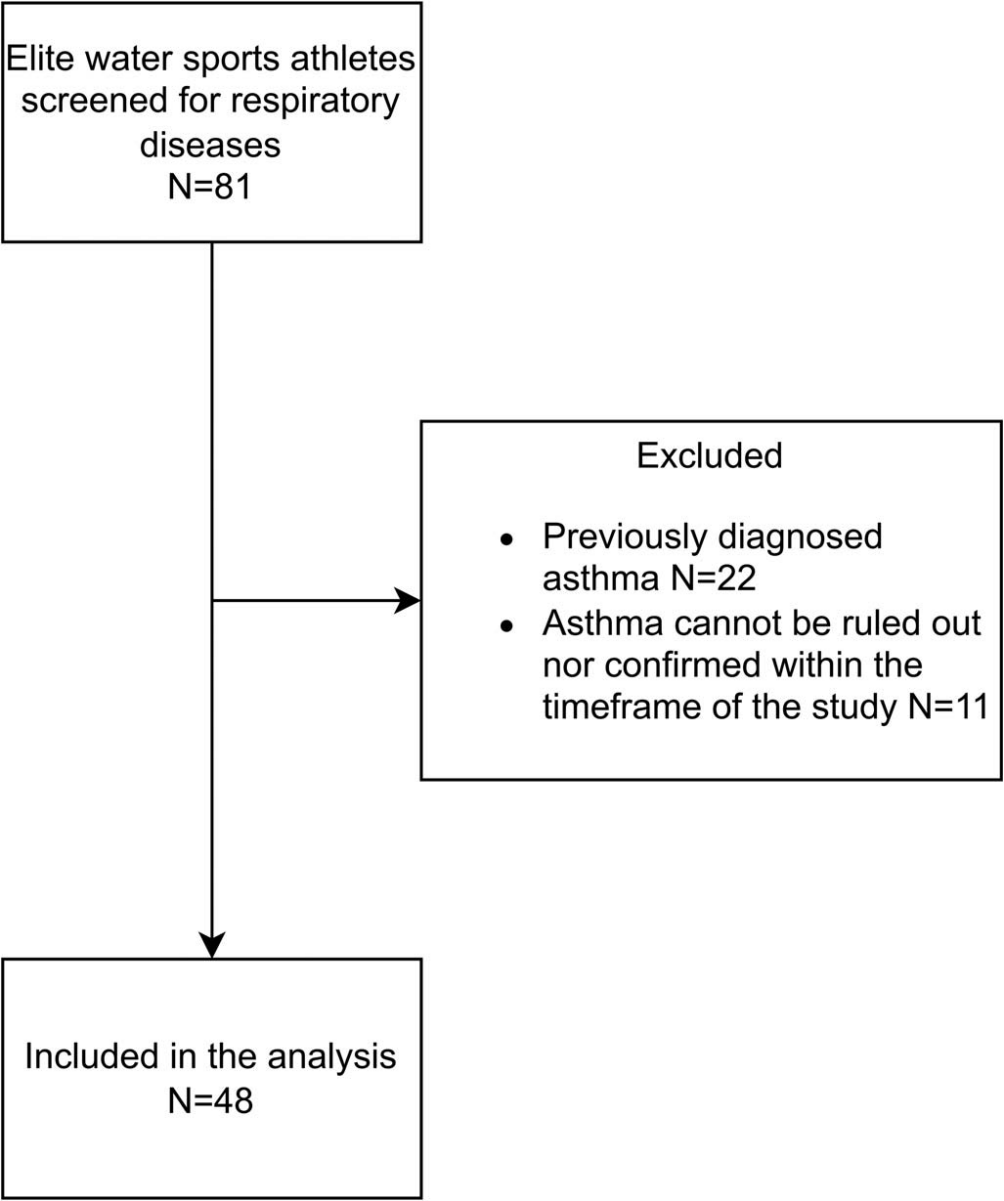
Flow chart of patient selection.

Exclusion criteria included previously diagnosed and treated bronchial asthma and a diagnosis of exercise-induced breathing disorders of different etiologies (e.g., exercise-induced laryngeal obstruction (17)). We also excluded participants for whom the diagnosis of asthma/EIB could not be ruled out or confirmed (e.g., atypical symptoms, indeterminate or borderline elevated biomarker levels, partial reversibility of airflow limitation [<12% but >200 mL]) at the end of the data collection period. In those participants, a longitudinal assessment was performed, which exceeded the timescale of our study. The participant selection process is illustrated in Figure 1.

### Design

This was a cross-sectional observational clinical study carried out at the Department of Pulmonology, Semmelweis University, Budapest, Hungary, between March 2021 and June 2022.

First, a thorough clinical assessment was performed, including assessment of clinical history, standard physical examination, the nature of the respiratory symptoms, and their relationship with exercise. Furthermore, participants underwent baseline investigations in the following sequence: venous blood sampling, F_ENO_ measurement, spirometry and body plethysmography with reversibility testing, and skin prick test. Further investigations (e.g., bronchoprovocation testing and peak expiratory flow [PEF] monitoring) were performed if the baseline spirometry and reversibility results were not suggestive of airway obstruction, but the participant had a history of exercise-induced respiratory symptoms and was clinically well at the time. Based on thorough clinical history, clinical signs and symptoms, results of the investigations, and a robust evaluation of the likelihood of the diagnosis, the diagnosis of asthma/EIB was confirmed by a highly trained senior respiratory specialist with experience in sports medicine (5,14,18,19).

The clinical characteristics and results of the investigations were compared between the asthmatic and the nonasthmatic participants. The diagnostic accuracy of BEC, F_ENO_, total IgE level, and prick test positivity was assessed. BEC, F_ENO_, and IgE concentrations were evaluated both as continuous and as dichotomized variables with the following predefined cutoff values: BEC, ≥150 cells per microliter; total IgE concentration, ≥100 IU·mL^−1^; and F_ENO_, ≥25 ppb (20–23). We developed a scoring system to predict asthma likelihood using dichotomous variables. Each variable was defined as either normal or increased based on the above cutoffs, and we assigned a score of 1 if the variable is elevated and 0 if it is not. These scores were summed to produce a final score ranging from 0 to 4.

The study was designed in accordance with the 1964 Declaration of Helsinki and its later amendments, and the protocol was approved by the ethics committee of the Semmelweis University Regional and Institutional Committee of Science and Research Ethics (approval no. 55263-6/2020/EÜIG).

### Methods of measurements

Venous blood samples were collected from all participants to measure the full blood count, including eosinophil granulocyte counts and percentages, and serum total IgE concentrations (Sysmex XN-1000, Sysmex Corporation, Kobe, Japan, and Beckman Coulter AU680, Beckman Coulter Inc., Indianapolis, IN).

Skin prick test was conducted according to international guidelines (24,25) by a trained healthcare professional. Participants taking oral antihistamine medications were instructed to stop the medication 5 d before the test. Histamine dihydrochloride served as a positive control, and physiological saline solution was used as a negative control. The tests were interpreted 15–20 min after application, and a positive result was defined as a wheal ≥3 mm in diameter. The following inhaled allergens were tested: *Dermatophagoides pteronyssinus*, *Dermatophagoides farinae*, *Cladosporium herbarum, Aspergillus fumigatus, Alternaria alternata*, dog dander, cat dander, mixed feathers, mixed trees (early and late blossom), grass mix, weed mix, cereal mix, mugwort, and common ragweed (Inmunotek Standard Prick Test Panel for Inhalants, Spiromed Ltd., Budapest, Hungary).

F_ENO_ was measured by a trained specialist using a chemiluminescent NO analyzer (Sievers NOA-280i, GE Analytical Instruments, Boulder, CO) according to international guidelines (21), and as previously described (26). All participants were nonsmokers and were asked to refrain from consuming food or beverages, taking inhaled medications, or exercising for 1 h before measurement. Exhaled NO analysis was performed for all participants before spirometry. The ambient NO level at the time of testing was <5 ppb. During the measurement, the participants were instructed to inhale to near-total lung capacity (TLC) and then exhale in the device against a calibrated resistance with a constant flow rate of 50 mL·s^−1^ until a plateau was reached at the NO level for at least 3 s.

Lung function parameters were measured by an electronic spirometer and a body plethysmograph (PDD-301/s, Piston, Budapest, Hungary) according to the European Respiratory Society (ERS) and American Thoracic Society (ATS) guidelines (27). The registered parameters included forced vital capacity (FVC), forced expiratory volume in the first second of exhalation (FEV_1_), FEV_1_ and FVC ratio, forced expiratory flow between 25% and 75% of FVC (FEF_25–75_), TLC, residual volume (RV), RV and TLC ratio, and airway resistance (R_aw_). Three technically acceptable maneuvers were performed with a between-maneuver difference in FVC and FEV_1_ ≤ 150 mL, and the highest result was used. The diffusing capacity of carbon monoxide (CO) (DL_CO_) and lung volume-corrected diffusing capacity (KL_CO_) was measured using the single-breath method (PDD-301/s, Piston). Pulmonary function variables were expressed as percentages of the predicted values using the Global Lung Function Initiative reference equations (28). In cases of suspected asthma, the following tests were performed according to the Global Initiative for Asthma document and international guidelines: reversibility testing after 400 μg of salbutamol inhalation (positive if FEV_1_ increases >200 mL and >12% of the predicted value), indirect bronchial challenge test with hypertonic potassium chloride solution inhalation (positive if FEV_1_ falls >15% of the predicted value), and/or daily and pre- and postexercise PEF measurements (positive if variability is >20% over a 2-wk period) (5,14,29).

### Statistical analysis

TIBCO Statistica (version 13; TIBCO Software Inc., Palo Alto, CA) and IBM SPSS (version 28; IBM Corp., Armonk, NY) statistical software packages were used for data analysis. Continuous variables were compared using unpaired *t*-tests or Mann–Whitney *U*-tests according to the distribution of variables. Categorical variables were analyzed using the chi-squared test or Fisher exact test. Receiver operating characteristic (ROC) curves were generated, and the area under the curve (AUC) was calculated to assess the diagnostic accuracy of F_ENO_, BEC, total IgE measurements, skin prick test, and the composite variable generated from these variables after dichotomization. We used Youden’s index to determine the optimal cutoff values based on ROC analysis. The index is determined by calculating the point on the curve where the sum of sensitivity and 1 specificity is the highest (30). Furthermore, binomial logistic regression models were used to explore the variables that predicted the diagnosis of asthma.

## RESULTS

The detailed clinical and demographic characteristics of the participants are summarized in Table 1. A total of 48 participants were enrolled in the study, of which 22 (46%) were diagnosed with asthma at the end of the clinical investigation. There was no difference in age or sex distribution between the asthmatic and the nonasthmatic groups. Participants in the asthmatic group had lower FEV_1_% of the predicted value, FEV_1_/FVC ratio, FEF_25–75_ values, and increased R_aw_ compared with nonasthmatic participants. The diffusing capacity of asthmatic participants was lower than that of nonasthmatics. Furthermore, the change in FEV_1_, both in raw value and in percentage of the predicted value, was greater in the asthmatic group during reversibility testing. Forty-two participants had a reversibility testing, of which 6 (14%) showed a positive response. Ten participants had bronchial challenge, of which 7 (70%) exhibited a positive response.

**TABLE 1:** Demographic and clinical parameters.

Variables	Total, *N* = 48	No Asthma, *n* = 26	New Asthma, *n* = 22	*P* Value, No Asthma vs New Asthma
Age, yr	21 ± 4	21 ± 4	20 ± 4	0.755
Sex, male/female	20/28	9/17	11/11	0.381
Weight, kg	69.2 ± 12	66.1 ± 11	73.0 ± 13	**0.043**
Height, cm	176.0 ± 10	174.2 ± 11	178.1 ± 9	0.145
BMI	21.9 (21.1–23.5)	21.6 (21.1–22.1)	23.1 (21.2–24.5)	0.064
FEV_1_, L	4.6 ± 0.9	4.7 ± 1.0	4.5 ± 0.8	0.605
FEV_1_, % pred.	110.8 ± 13.8	116.0 ± 14.3	104.7 ± 10.5	**0.001**
∆FEV_1_, L	0.29 (0.14–0-47)	0.17 (0.08–0.32)	0.37 (0.25–0.54)	**0.005**
∆FEV_1_, % pred.	7 (3–9)	6 (2–8)	9 (4–13)	**0.035**
FVC, L	5.8 ± 1.3	5.7 ± 1.3	6.0 ± 1.3	0.341
FVC, % pred.	119.9 ± 14.7	120.3 ± 11.6	119.5 ± 18.0	0.764
FEV_1_/FVC	0.79 ± 0.07	0.81 ± 0.06	0.76 ± 0.06	**0.003**
FEF_25–75_, L	4.34 ± 1.0	4.67 ± 1.0	3.94 ± 0.8	**0.007**
FEF_25–75_, % pred.	93.7 ± 19.0	103.0 ± 16.7	82.6 ± 15.4	**<0.001**
R_aw_, kPa*s·L^−1^	0.25 ± 0.07	0.22 ± 0.05	0.27 ± 0.08	**0.018**
R_aw_, % pred.	115.5 ± 47.7	84.7 ± 21.5	125.8 ± 50.4	0.114
Dl_CO_, % pred.	149.0 ± 22.4	155.2 ± 20.3	140.4 ± 22.9	**0.042**
Kl_CO_, % pred.	122.0 ± 21.4	125.8 ± 19.2	116.8 ± 23.5	0.179
WBC, 10^9^·L^−1^	6.7 ± 2.0	6.4 ± 1.6	7.1 ± 2.3	0.273
Eosinophil granulocyte count, cells per microliter	120 (80–190)	140 (80–190)	110 (80–190)	0.901
Eosinophil granulocyte, % of WBC	1.9 (1.1–3.4)	1.9 (1.1–3.4)	2.1 (1.1–3.4)	0.992
Total IgE, IU·mL^−1^	30.2 (14.5–113.4)	24.1 (11.2–43.9)	67.7 (27.4–175.8)	**0.011**
F_ENO_, ppb	18 (14–25)	15 (11–22)	20 (17–26)	**0.020**
Positive prick test, *n* (%)	14 (30)	2 (8)	12 (55)	**0.001**

Data are presented as mean ± SD or median (interquartile range) and are compared with unpaired *t*-test or Mann–Whitney *U*-test. Statistically significant differences (*P* < 0.05) are highlighted in bold.

BMI, body mass index; DL_CO_, diffusing capacity for carbon monoxide; FEF_25–75_, forced expiratory flow between 25% and 75% of FVC; FEV_1_, forced expiratory volume in the first second; ∆FEV_1_, change in FEV_1_ during reversibility testing (% change representing absolute change); IU, international unit; KL_CO_, transfer coefficient of the lung for carbon monoxide; ppb, parts per billion; WBC, white blood cell count.

**FIGURE 2:**
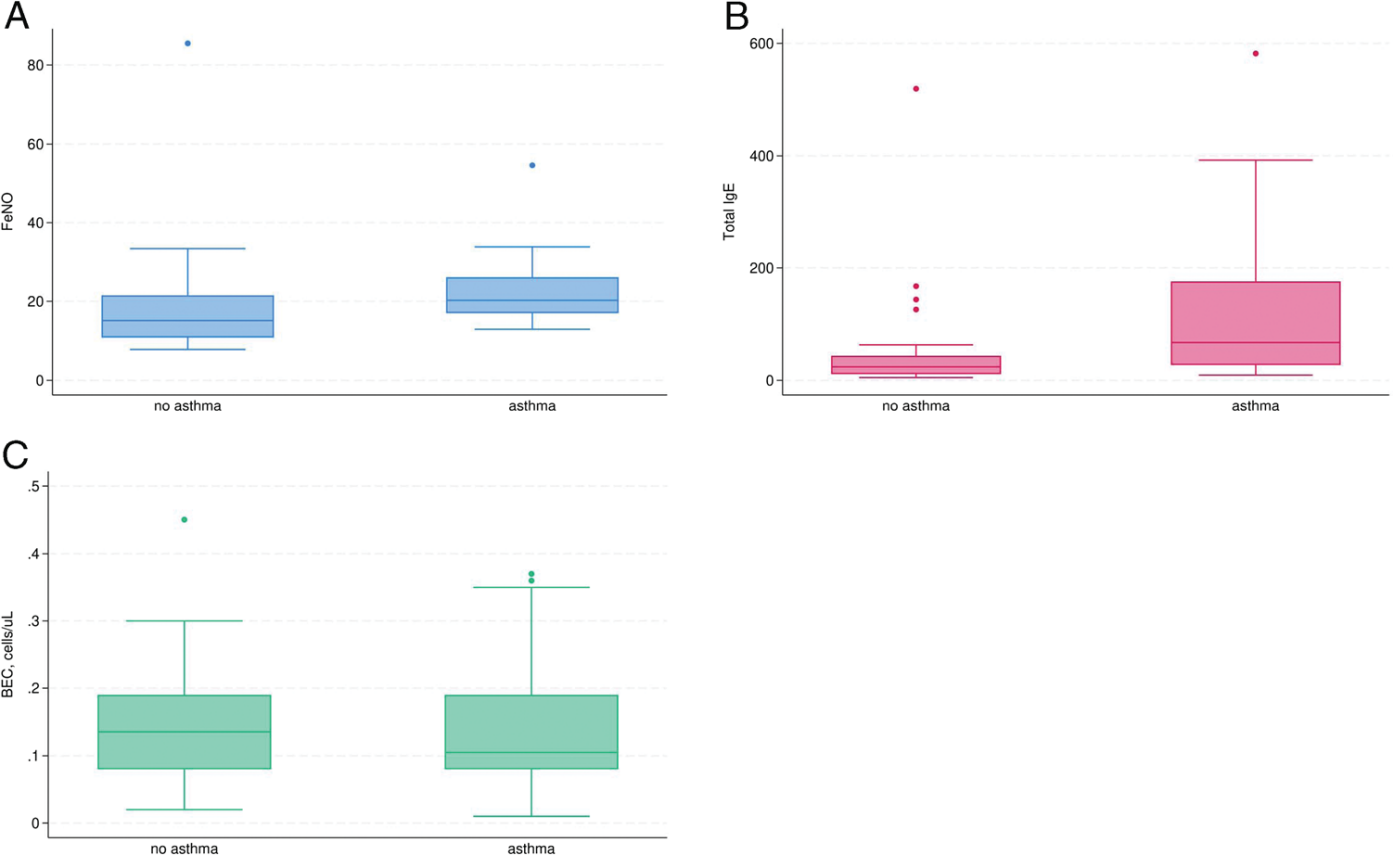
Levels of F_ENO_ (A), IgE (B), and BEC (C) in nonasthmatic and asthmatic participants.

There were no differences in BEC or eosinophil granulocyte/total white blood cell count ratios between the two groups. The total IgE and F_ENO_ concentrations were elevated in asthmatic participants compared with those in the nonasthmatic group (24.1 [11.2–43.9] vs 67.7 [27.4–175.8] IU·mL^−1^; 15 [11–22] vs 20 [17–26] ppb; Figure 2). The ratio of prick test positivity was also higher in the asthmatic group.

**FIGURE 3:**
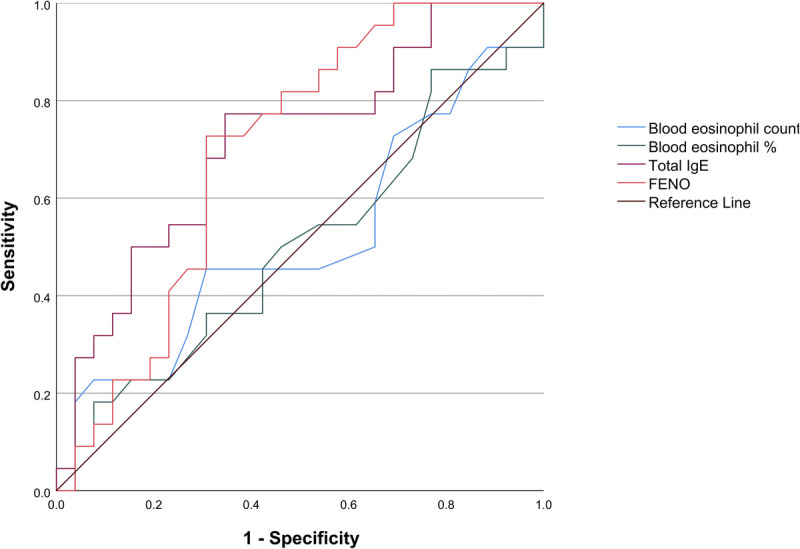
ROC curves of BEC, blood eosinophil percentage, total IgE concentration, and F_ENO_ to predict asthma diagnosis.

The ROC curves of the individual biomarkers are shown in Figure 3. BEC and eosinophil granulocyte percentages had an area under the ROC curve of 0.51 (95% confidence interval [CI] = 0.34–0.68) and 0.50 (95% CI = 0.33–0.67), respectively. Total IgE and F_ENO_ concentrations were suitable for discriminating asthmatic participants from nonasthmatic individuals with AUC values of 0.72 (95% CI = 0.57–0.86, *P* = 0.01) and 0.70 (95% CI = 0.55–0.85, *P* = 0.02). We calculated the optimal cutoff values of IgE and F_ENO_ based on Youden’s index to balance sensitivity and specificity. An IgE cutoff of 30 IU·mL^−1^ yielded a sensitivity of 77% and a specificity of 65%. A F_ENO_ cutoff of 20 ppb had a sensitivity of 72% and specificity of 65%.

We created a composite variable from the BEC, total IgE, F_ENO_, and prick test results. BEC, total IgE, and F_ENO_ levels were dichotomized with cutoff values of ≥150 cells per microliter, ≥100 IU·mL^−1^, and ≥25 ppb, respectively. Each component was given a score of 0/1, and the sum of the scores was used as an ordinal variable. Asthmatic participants had significantly higher scores than nonasthmatics (2 [1–3] vs 1 [0–1], Mann–Whitney *U*-test *P* = 0.02). ROC analysis of the scoring system was performed. The composite biomarker variable could distinguish between the asthmatic and the nonasthmatic participants, with an AUC of 0.69 (95% CI = 0.53–0.85, *P* = 0.03). The best cutoff value to balance sensitivity and specificity was 2 points, with a sensitivity of 54.5%, a specificity of 80.8%, a positive predictive value (PPV) of 70.6%, a negative predictive value (NPV) of 67.7%, and a likelihood ratio of 2.8. However, a cutoff point of 3 achieved a specificity of 96.2%, a PPV of 88.9%, and a likelihood ratio of 9.5, meaning that a result of >2 points gives a very strong prediction of asthma in this population (Table 2). However, it is important to note that the sensitivity at this cutoff remains relatively low at 36.4%, with an NPV of 64.1%.

**TABLE 2:** Cross-tabulation of the proposed scoring system and positive asthma diagnosis.

	No Asthma	Asthma	Total
Composite score ≤2	25	14	39
Composite score >2	1	8	9
Total	26	22	48

Fisher exact test *P* < 0.01.

Binary logistic regression was performed to assess the effects of sex, weight, baseline FEV_1_%, and the composite biomarker variable on the likelihood of having asthma (Table 3). The regression model was statistically significant (chi-square = 14.5, *P* = 0.04), explained 52.4% of the variance in asthma diagnosis (Nagelkerke *R*^2^ = 0.54), and correctly classified 84.8% of cases. Each score increment in the composite variable increased the likelihood of asthma by 2.7 times. Increased weight was associated with an increased likelihood of asthma. The baseline FEV_1_% was negatively associated with asthma. Sex did not significantly contribute to the model. The individual components of the composite variable were not tested in the model because they are not independent (i.e., they share a common pathophysiological background), and it would violate the assumptions of the binary logistic regression. Age was excluded from the model because the age of the participants was homogenous and all were young adults; therefore, it had no clinical relevance.

**TABLE 3:** Results of binary logistic regression model.

	Odds Ratio	95% CI for the Odds Ratio	*P* Value (Wald Test)
Sex (female)	0.21	0.02–2.14	0.188
Weight	1.11	1.01–1.23	**0.031**
Baseline FEV_1_, % pred.	0.90	0.82–0.98	**0.017**
Composite biomarker variable	2.71	1.17–6.23	**0.020**

*P* values <0.05 are highlighted in bold. The composite biomarker variable is produced from sum of the dichotomized values of BEC, total IgE concentration, F_ENO_, and positive prick test.

FEV_1_, forced expiratory volume in the first second of exhalation.

## DISCUSSION

To the best of our knowledge, this is the first clinical study designed to evaluate a complex biomarker assessment for diagnosing bronchial asthma in a cohort of elite water sports athletes. We have proposed an easy-to-use scoring system based on Th2-type inflammation markers for elite water sports athletes with respiratory symptoms, which is strongly associated with a positive diagnosis of asthma and may therefore aid in the diagnosis. However, it is essential to acknowledge the limitation of our proposed diagnostic method, i.e., its low sensitivity and negative predictive value. Furthermore, we have confirmed the previously recognized high prevalence of asthma within this population.

Lower airway dysfunction is common in the general population and are more frequent in elite athletes. Levai et al. (31) found that the prevalence of EIB measured by indirect bronchial provocation tests can reach almost 70% in elite swimmers. Meanwhile, a large-scale international study conducted by collecting therapeutic use exemptions data of elite competitive athletes found that the combined prevalence of asthma and airway hyperresponsiveness is around 22% in aquatic disciplines (7). The huge discrepancies in reported prevalence may be explained by the different study designs (performing new measurements vs collecting existing data), sample sizes, and assessed population (national vs international). Nevertheless, a recent systematic review and meta-analysis summarizing the results of studies involving more than 37,000 participants concluded the prevalence of lower airway disfunction in water sports athletes lies around 40% (11). The reason for the high prevalence of lower airway pathologies in aquatic sports is mainly thought to be caused by the chronic inhalation of volatile chlorine by-products, e.g., trichloramines originating from the disinfection of water pools (32) and by repeated airway epithelial microinjuries caused by sheer stress due to high minute ventilation (33). However, numerous studies have attempted to link chlorine products to the increased prevalence of asthma among swimmers, with conflicting results (32,34,35). Bougault et al. (36) collected respiratory tissue samples from nonasthmatic elite swimmers, asthmatic nonswimmers, and nonasthmatic nonathletes. They found that swimmers had inflammatory and remodeling changes similar to asthmatics and concluded that this was due to long-term exposure to chlorinated swimming pools. On the other hand, Llana-Belloch et al. (34) did not find markers of increased oxidative stress or lung damage after 40 min of exercise in indoor swimming pools despite significant exposure to chlorine by-products. Nevertheless, it is likely that chlorine by-products, mainly trichloramines, act as sensitizers against airborne allergens, leading to the development of atopy and airway remodeling (7,31,36–38). However, the increased prevalence of exercise-induced airway pathologies in swimmers is likely due to a complex mechanism involving genetic and epigenetic predisposition, airway inflammation and remodeling caused by recurrent viral respiratory infections, and chronic epithelial damage and sensitization from exposure to chlorine by-products (32,39). Regardless of the underlying cause, asthma-specific, mainly Th2-driven, inflammation is present in this population as well, and certain markers of Th2 inflammation may aid in the diagnosis and monitoring of the disease.

However, is it paramount to acknowledge that more than 60% of the newly diagnosed asthmatic individuals did not have Th2-type inflammation as assessed by our composite variable, and it limits the applicability of our proposed scoring system. Indeed, Couto et al. (40) have proven by latent class analysis that there are two types of asthma in athletes, one is the classical “atopic asthma,” driven by Th2-type inflammation, and the other is a non-Th2-type disease, which the authors call “sports asthma,” defined by exercise-induced airway symptoms without features of atopic allergy. Importantly, sports asthma is not associated with elevated F_ENO_ or other classic markers of asthma and is more common in aquatic and winter sports. Couto et al. have found that participation in competitive water sports was associated with an almost threefold increase in the odds of having this type of disease, whereas winter sports athletes had an odds ratio of more than 8.5 compared with other athletes. Several mechanisms have been proposed that may lead to the development of sports asthma (33,41). For example, dehydration due to thermal changes and increased ventilation and consequent osmotic changes may lead to bronchoconstriction via bronchial wall edema and airway smooth muscle contraction through the activation of inflammatory pathways and changes in airway vascular diameter (33,41,42). However, dehydration seems unlikely in water sports athletes considering the humid environment above the water surface. In this population, the microtrauma theory is more likely, suggesting that athletes suffer repeated airway epithelial injuries due to the mechanical stress caused by high minute ventilation, exposure to chemicals, and microaspirations of water droplets (43). The repeated injury and healing process leads to altered contractile function of the small airways, contributing to the development of exercise-induced airway symptoms (44). This theory might be further supported by the observation that the inflammatory and remodeling processes may be reversed in some cases by quitting swimming (45). Nevertheless, the high prevalence of “sports asthma” most likely contributed to the low sensitivity and negative predictive value of our composite diagnostic test based on Th2-type biomarkers.

Nitric oxide is a gaseous molecule that can be measured in the exhaled breath (21). The fraction of exhaled NO (F_ENO_) is associated with eosinophilic airway inflammation and is therefore an important biomarker of bronchial asthma (14). However, significant intra- and interindividual variation can be observed in the levels of F_ENO_, which necessitates the setup and use of a standardized and reliable measurement protocol, as endorsed by the ATS and the ERS (21). Accordingly, based on the high variability and the observation that F_ENO_ can be elevated in conditions other than asthma, the Global Initiative for Asthma document does not recommend the use of F_ENO_ for ruling in or ruling out the diagnosis of asthma (29). Nevertheless, the diagnostic usefulness of F_ENO_ is recognized by the ERS in their most recent asthma diagnosis guideline and by the clinical statement of the British Thoracic Society (BTS) addressing the diagnosis, evaluation, and management of respiratory problems in athletic individuals (5,14). The ERS recommends a cutoff of >50 ppb for a specificity of >90% or a cutoff of >40 ppb for the best compromise between sensitivity and specificity. In support of this, Dickinson et al. (16) have reported a study involving recreational and elite athletes from a wide range of athletic disciplines, that F_ENO_ ≥ 40 ppb predicts EIB with the best specificity/sensitivity ratio (86% and 37%, respectively). However, given the low sensitivity, the authors recommend against using F_ENO_ alone in diagnosing EIB. Similarly, the ERS guideline emphasizes that all biomarkers, including F_ENO_, must be interpreted in the context of the symptoms and the clinical picture, and a low F_ENO_ level does not rule out asthma. The BTS recommends measuring F_ENO_ as part of the diagnostic workup in athletes, but they do not provide instructions about the interpretation of the results. However, the ATS clinical practice guideline for the interpretation of F_ENO_ defines high, intermediate, and low levels, with cutoffs of >50, 25–50, and <25 ppb, respectively (20). Consequently, we selected a cutoff of ≥25 ppb for our composite variable to include individuals even within the intermediate F_ENO_ level to ensure that we did not exclude anyone with eosinophilic airway inflammation. This choice was driven by the fact that our study focused on elite athletes who engage in extreme physical performance, and even minor disturbances may have a significant impact on performance. Moreover, our findings indicate that F_ENO_ levels are elevated in individuals with asthma, and the Youden’s index based on the ROC analysis determined an optimal cutoff point for F_ENO_ at 20 ppb for predicting the diagnosis of asthma. Although it is lower than that recommended in the ERS guidelines, this cutoff was calculated to optimize the sensitivity and specificity, whereas the guideline’s higher cutoff was fitted to yield a high specificity to confidently rule in the diagnosis of asthma in symptomatic participants. Our F_ENO_ results and the calculated cutoff are in line with a series of previous studies (46–49). Nevertheless, designing prospective studies to assess other cutoff values of F_ENO_ for diagnosing asthma in elite swimmers may be of interest.

Allergy is the most common pathophysiological characteristic of asthma in the general population, almost 60% of asthma cases are attributable to atopy, according to a nationwide survey conducted in the United States involving more than 12,000 participants (50). In the pathogenesis of type 1 hypersensitivity, IgE plays a critical role in mediating the degranulation of mast cells and basophils and subsequently leads to the recruitment and activation of eosinophils (51). The level of total serum IgE correlates with asthma in adults and pediatric patients, as has been proven in large-scale international studies (52,53). We also found that IgE levels were elevated in asthmatic participants compared with nonasthmatic participants and that it could distinguish between those with and without asthma. However, our cutoff value calculated using Youden’s index was lower than that reported previously (48,54). Furthermore, the skin prick test is also associated with allergic asthma, and we found that more than half of the asthmatic participants were sensitized to at least one inhaled allergen. This is in line with the results of the Third National Health and Nutrition Examination Survey in the United States, which found that 56.3% of patients with asthma also had atopy (50).

Eosinophil granulocytes are important participators of allergic inflammation, and their role in the pathogenesis of asthma, i.e., in airway remodeling, has been proven long ago (55). Identifying eosinophilic airway inflammation is possible through the analysis of induced sputum or bronchoalveolar lavage, but it is burdensome to both the patient and the healthcare system and is thus rarely feasible (56). However, BEC may reflect the airway compartment, and its measurement is widely accessible. Furthermore, according to large-scale epidemiological studies, elevated BEC is associated with poor disease outcomes and frequent exacerbations (57). Therefore, several tailored biological treatments of asthma aim to lower BEC and thus ameliorate eosinophilic inflammation through targeting key inflammatory cytokines, e.g., interleukin (IL)-5, IL-4, and IL-13 (29,58). However, we did not find differences in either the absolute eosinophil count or eosinophil ratios between asthmatic and nonasthmatic participants. A possible explanation may be that our study did not have a sufficient sample size to detect the difference because according to a study involving more than 130,000 asthmatic patients, only about one-fifth of the patients present with elevated BEC (57). Furthermore, swimming may also influence airway eosinophils, although existing research on this matter has shown mixed results. Bougault et al. (36,59) reported elevated eosinophil counts in induced sputum and bronchoscopic samples of nonasthmatic swimmers, whereas others found no significant differences between swimmers and nonswimmers (60,61). However, the evidence suggests that swimming may not significantly impact BEC levels (62,63).

The strength of our study is that we were able to enroll the majority of the National Swim Team of Hungary, which allowed us to perform the analyses with a sufficient sample size to draw certain conclusions about this highly specific population. Furthermore, our scoring system showed a fairly strong association with the diagnosis of asthma, and the required tests are widely accessible in secondary care and sometimes even in primary care. However, our study has limitations as well. First, because of the single-center setting and the single ethnicity of our population, our results may not be universal and need further validation in multicenter studies with the enrolment of mixed ethnicities. Furthermore, although the sample size is substantial considering the relatively small population of elite water sports athletes in our region, it may still be insufficient to comprehensively explore various associations beyond the primary focus of our study. Moreover, we are also aware that the ERS guideline recommends against the combined use of F_ENO_, BEC, and serum IgE in the diagnosis of asthma as the accuracy of the combined test may not be substantially higher than of the individual tests; however, their recommendation is conditional and is based on one single-center study that assessed a different population (14). Additionally, we involved in our combined system the skin prick test, which may increase the diagnostic accuracy.

## CONCLUSIONS

In this study, we investigated the prevalence of asthma in elite water sports athletes. Our findings confirmed a high prevalence of asthma in this population, emphasizing the need for systematic expert evaluation to avoid underdiagnosis. Additionally, we introduced an easy-to-use scoring system based on Th2-type inflammation markers. This scoring system showed a strong association with a diagnosis of asthma. Further prospective studies are needed to validate the scoring system as a diagnostic tool for elite water sports athletes with respiratory symptoms.

## References

[1] GBD 2019 Diseases and Injuries Collaborators. Global burden of 369 diseases and injuries in 204 countries and territories, 1990–2019: a systematic analysis for the Global Burden of Disease Study 2019. *Lancet*. 2020;396(10258):1204–22. 33069326 10.1016/S0140-6736(20)30925-9 PMC7567026

[2] Weiler JM Brannan JD Randolph CC, . Exercise-induced bronchoconstriction update—2016. *J Allergy Clin Immunol*. 2016;138(5):1292–1295.e36. 27665489 10.1016/j.jaci.2016.05.029

[3] Sonna LA Angel KC Sharp MA Knapik JJ Patton JF Lilly CM. The prevalence of exercise-induced bronchospasm among US Army recruits and its effects on physical performance. *Chest*. 2001;119(6):1676–84. 11399690 10.1378/chest.119.6.1676

[4] Ng'ang'a LW Odhiambo JA Mungai MW, . Prevalence of exercise induced bronchospasm in Kenyan school children: an urban-rural comparison. *Thorax*. 1998;53(11):919–26. 10193388 10.1136/thx.53.11.919 PMC1745121

[5] Hull JH Burns P Carre J, . BTS clinical statement for the assessment and management of respiratory problems in athletic individuals. *Thorax*. 2022;77(6):540–51. 35410960 10.1136/thoraxjnl-2021-217904

[6] Bonini M Gramiccioni C Fioretti D, . Asthma, allergy and the Olympics: a 12-year survey in elite athletes. *Curr Opin Allergy Clin Immunol*. 2015;15(2):184–92. 25961393 10.1097/ACI.0000000000000149

[7] Mountjoy M Fitch K Boulet L-P Bougault V van Mechelen W Verhagen E. Prevalence and characteristics of asthma in the aquatic disciplines. *J Allergy Clin Immunol*. 2015;136(3):588–94. 25819982 10.1016/j.jaci.2015.01.041

[8] Boulet LP O'Byrne PM. Asthma and exercise-induced bronchoconstriction in athletes. *N Engl J Med*. 2015;372(7):641–8. 25671256 10.1056/NEJMra1407552

[9] Hostrup M Hansen ESH Rasmussen SM Jessen S Backer V. Asthma and exercise-induced bronchoconstriction in athletes: diagnosis, treatment, and anti-doping challenges. *Scand J Med Sci Sports*. 2024;34(1):e14358. 36965010 10.1111/sms.14358

[10] Price OJ Hull JH Backer V Hostrup M Ansley L. The impact of exercise-induced bronchoconstriction on athletic performance: a systematic review. *Sports Med*. 2014;44(12):1749–61. 25129699 10.1007/s40279-014-0238-y

[11] Price OJ Sewry N Schwellnus M, . Prevalence of lower airway dysfunction in athletes: a systematic review and meta-analysis by a subgroup of the IOC consensus group on 'acute respiratory illness in the athlete'. *Br J Sports Med*. 2022;56(4):213–22. 34872908 10.1136/bjsports-2021-104601

[12] Mazic S Lazovic B Djelic M, . Respiratory parameters in elite athletes—does sport have an influence? *Rev Port Pneumol (2006)*. 2015;21(4):192–7. 25926244 10.1016/j.rppnen.2014.12.003

[13] Reier-Nilsen T Sewry N Chenuel B, . Diagnostic approach to lower airway dysfunction in athletes: a systematic review and meta-analysis by a subgroup of the IOC consensus on 'acute respiratory illness in the athlete'. *Br J Sports Med*. 2023;57(8):481–9. 36717213 10.1136/bjsports-2022-106059

[14] Louis R Satia I Ojanguren I, . European Respiratory Society guidelines for the diagnosis of asthma in adults. *Eur Respir J*. 2022;2101585. 35169025 10.1183/13993003.01585-2021

[15] Lázár Z Kelemen A Gálffy G Losonczy G Horváth I Bikov A. Central and peripheral airway nitric oxide in patients with stable and exacerbated chronic obstructive pulmonary disease. *J Breath Res*. 2018;12(3):036017. 29813036 10.1088/1752-7163/aac10a

[16] Dickinson J Gowers W Sturridge S, . Fractional exhaled nitric oxide in the assessment of exercise-induced bronchoconstriction: a multicenter retrospective analysis of UK-based athletes. *Scand J Med Sci Sports*. 2023;33(7):1221–30. 37051807 10.1111/sms.14367

[17] Clemm HH Olin JT McIntosh C, . Exercise-induced laryngeal obstruction (EILO) in athletes: a narrative review by a subgroup of the IOC consensus on 'acute respiratory illness in the athlete'. *Br J Sports Med*. 2022;56(11):622–9. 35193856 10.1136/bjsports-2021-104704 PMC9120388

[18] World Anti Doping Agency, *TUE Physician Guidelines—Asthma*. 2023. Available at https://www.wada-ama.org/en/resources/therapeutic-use-exemption/tue-physician-guidelines-asthma.

[19] Schwellnus M Adami PE Bougault V, . International Olympic Committee (IOC) consensus statement on acute respiratory illness in athletes part 2: non-infective acute respiratory illness. *Br J Sports Med*. 2022;bjsports-2022-105567. 10.1136/bjsports-2022-105567 35623888

[20] Dweik RA Boggs PB Erzurum SC, . An official ATS clinical practice guideline: interpretation of exhaled nitric oxide levels (FENO) for clinical applications. *Am J Respir Crit Care Med*. 2011;184(5):602–15. 21885636 10.1164/rccm.9120-11ST PMC4408724

[21] American Thoracic Society; European Respiratory Society. ATS/ERS recommendations for standardized procedures for the online and offline measurement of exhaled lower respiratory nitric oxide and nasal nitric oxide, 2005. *Am J Respir Crit Care Med*. 2005;171(8):912–30. 15817806 10.1164/rccm.200406-710ST

[22] Yancey SW Keene ON Albers FC, . Biomarkers for severe eosinophilic asthma. *J Allergy Clin Immunol*. 2017;140(6):1509–18. 29221581 10.1016/j.jaci.2017.10.005

[23] Johns CB Laidlaw TM. Elevated total serum IgE in nonatopic patients with aspirin-exacerbated respiratory disease. *Am J Rhinol Allergy*. 2014;28(4):287–9. 25197914 10.2500/ajra.2014.28.4054

[24] Bousquet J Heinzerling L Bachert C, . Practical guide to skin prick tests in allergy to aeroallergens. *Allergy*. 2012;67(1):18–24. 22050279 10.1111/j.1398-9995.2011.02728.x

[25] Heinzerling L Mari A Bergmann K-C, . The skin prick test—European standards. *Clin Transl Allergy*. 2013;3(1):3. 23369181 10.1186/2045-7022-3-3 PMC3565910

[26] Csoma B Beringer F Szűcs G Bikov A Müller V Lázár Z. Measurements of upper and lower airway nitric oxide in healthy adults. *J Breath Res*. 2021;15(4):041002. 10.1088/1752-7163/ac2567 34500438

[27] Graham BL Steenbruggen I Miller MR, . Standardization of spirometry 2019 update. An official American Thoracic Society and European Respiratory Society technical statement. *Am J Respir Crit Care Med*. 2019;200(8):e70–88. 31613151 10.1164/rccm.201908-1590ST PMC6794117

[28] Quanjer PH Stanojevic S Cole TJ, . Multi-ethnic reference values for spirometry for the 3–95-yr age range: the global lung function 2012 equations. *Eur Respir J*. 2012;40(6):1324–43. 22743675 10.1183/09031936.00080312 PMC3786581

[29] Global Initiative for Asthma. Global strategy for asthma management and prevention. 2021. Available from: www.ginasthma.org.

[30] Youden WJ. Index for rating diagnostic tests. *Cancer*. 1950;3(1):32–5. 15405679 10.1002/1097-0142(1950)3:1<32::aid-cncr2820030106>3.0.co;2-3

[31] Levai IK Hull JH Loosemore M Greenwell J Whyte G Dickinson JW. Environmental influence on the prevalence and pattern of airway dysfunction in elite athletes. *Respirology*. 2016;21(8):1391–6. 27460127 10.1111/resp.12859

[32] Bougault V Boulet L-P. Is there a potential link between indoor chlorinated pool environment and airway remodeling/inflammation in swimmers? *Expert Rev Respir Med*. 2012;6(5):469–71. 23134238 10.1586/ers.12.51

[33] Couto M Kurowski M Moreira A, . Mechanisms of exercise-induced bronchoconstriction in athletes: current perspectives and future challenges. *Allergy*. 2018;73(1):8–16. 28599081 10.1111/all.13224

[34] Llana-Belloch S Priego Quesada JI Pérez-Soriano P, . Disinfection by-products effect on swimmers oxidative stress and respiratory damage. *Eur J Sport Sci*. 2016;16(5):609–17. 26364906 10.1080/17461391.2015.1080306

[35] Kanikowska A Napiórkowska-Baran K Graczyk M Kucharski MA. Influence of chlorinated water on the development of allergic diseases—an overview. *Ann Agric Environ Med*. 2018;25(4):651–5. 30586974 10.26444/aaem/79810

[36] Bougault V Loubaki L Joubert P, . Airway remodeling and inflammation in competitive swimmers training in indoor chlorinated swimming pools. *J Allergy Clin Immunol*. 2012;129(2):351–8, 358.e1. 22196771 10.1016/j.jaci.2011.11.010

[37] Bougault V Boulet L-P. Airways disorders and the swimming pool. *Immunol Allergy Clin North Am*. 2013;33(3):395–408 ix. 23830132 10.1016/j.iac.2013.02.008

[38] Jacobs JH Spaan S van Rooy GBGJ, . Exposure to trichloramine and respiratory symptoms in indoor swimming pool workers. *Eur Respir J*. 2007;29(4):690–8. 17107995 10.1183/09031936.00024706

[39] Schwellnus M Adami PE Bougault V, . International Olympic Committee (IOC) consensus statement on acute respiratory illness in athletes part 1: acute respiratory infections. *Br J Sports Med*. 2022;bjsports-2022-105759. 10.1136/bjsports-2022-105759 35863871

[40] Couto M Stang J Horta L, . Two distinct phenotypes of asthma in elite athletes identified by latent class analysis. *J Asthma*. 2015;52(9):897–904. 26377281 10.3109/02770903.2015.1067321

[41] Rasmussen SM Hansen ESH Backer V. Asthma in elite athletes—do they have type 2 or non-type 2 disease? A new insight on the endotypes among elite athletes. *Front Allergy*. 2022;3:973004. 36340019 10.3389/falgy.2022.973004 PMC9633848

[42] Ali Z Norsk P Ulrik CS. Mechanisms and management of exercise-induced asthma in elite athletes. *J Asthma*. 2012;49(5):480–6. 22515573 10.3109/02770903.2012.676123

[43] Haahtela T Malmberg P Moreira A. Mechanisms of asthma in Olympic athletes—practical implications. *Allergy*. 2008;63(6):685–94. 18445185 10.1111/j.1398-9995.2008.01686.x

[44] Anderson SD Kippelen P. Airway injury as a mechanism for exercise-induced bronchoconstriction in elite athletes. *J Allergy Clin Immunol*. 2008;122(2):225–35 quiz 236-7. 18554705 10.1016/j.jaci.2008.05.001

[45] Bougault V Odashiro P Turmel J, . Changes in airway inflammation and remodelling in swimmers after quitting sport competition. *Clin Exp Allergy*. 2018;48(12):1748–51. 30141830 10.1111/cea.13257

[46] Fortuna AM Feixas T González M Casan P. Diagnostic utility of inflammatory biomarkers in asthma: exhaled nitric oxide and induced sputum eosinophil count. *Respir Med*. 2007;101(11):2416–21. 17714927 10.1016/j.rmed.2007.05.019

[47] Arora R Thornblade CE Dauby P-AL Flanagan JW Bush AC Hagan LL. Exhaled nitric oxide levels in military recruits with new onset asthma. *Allergy Asthma Proc*. 2006;27(6):493–8. 17176784 10.2500/aap.2006.27.2904

[48] Nekoee H Graulich E Schleich F, . Are type-2 biomarkers of any help in asthma diagnosis? *ERJ Open Res*. 2020;6(2):00169–2020. 10.1183/23120541.00169-2020 PMC7369447 32714964

[49] Malinovschi A Backer V Harving H Porsbjerg C. The value of exhaled nitric oxide to identify asthma in smoking patients with asthma-like symptoms. *Respir Med*. 2012;106(6):794–801. 22405608 10.1016/j.rmed.2012.02.009

[50] Arbes SJ Jr. Gergen PJ Vaughn B Zeldin DC. Asthma cases attributable to atopy: results from the Third National Health and Nutrition Examination Survey. *J Allergy Clin Immunol*. 2007;120(5):1139–45. 17889931 10.1016/j.jaci.2007.07.056 PMC2291202

[51] Moon TC Befus AD Kulka M. Mast cell mediators: their differential release and the secretory pathways involved. *Front Immunol*. 2014;5:569. 25452755 10.3389/fimmu.2014.00569 PMC4231949

[52] Burrows B Martinez FD Halonen M Barbee RA Cline MG. Association of asthma with serum IgE levels and skin-test reactivity to allergens. *N Engl J Med*. 1989;320(5):271–7. 2911321 10.1056/NEJM198902023200502

[53] Sears MR Burrows B Flannery EM Herbison GP Hewitt CJ Holdaway MD. Relation between airway responsiveness and serum IgE in children with asthma and in apparently normal children. *N Engl J Med*. 1991;325(15):1067–71. 1891008 10.1056/NEJM199110103251504

[54] Tilemann L Gindner L Meyer F Szecsenyi J Schneider A. Differences in local and systemic inflammatory markers in patients with obstructive airways disease. *Prim Care Respir J*. 2011;20(4):407–14. 21808940 10.4104/pcrj.2011.00069 PMC6549883

[55] Humbles AA Lloyd CM McMillan SJ, . A critical role for eosinophils in allergic airways remodeling. *Science*. 2004;305(5691):1776–9. 15375268 10.1126/science.1100283

[56] Walford HH Doherty TA. Diagnosis and management of eosinophilic asthma: a US perspective. *J Asthma Allergy*. 2014;7:53–65. 24748808 10.2147/JAA.S39119 PMC3990389

[57] Price DB Rigazio A Campbell JD, . Blood eosinophil count and prospective annual asthma disease burden: a UK cohort study. *Lancet Respir Med*. 2015;3(11):849–58. 26493938 10.1016/S2213-2600(15)00367-7

[58] Bardin PG Foster PS. Clinical translation of basic science in asthma. *N Engl J Med*. 2021;385(18):1714–7. 34706176 10.1056/NEJMe2114472

[59] Bougault V Turmel J St-Laurent J Bertrand M Boulet L-P. Asthma, airway inflammation and epithelial damage in swimmers and cold-air athletes. *Eur Respir J*. 2009;33(4):740–6. 19129276 10.1183/09031936.00117708

[60] Belda J Ricart S Casan P, . Airway inflammation in the elite athlete and type of sport. *Br J Sports Med*. 2008;42(4):244–8 discussion 248-9. 17711871 10.1136/bjsm.2007.036335

[61] Martin N Lindley MR Hargadon B Monteiro WR Pavord ID. Airway dysfunction and inflammation in pool- and non-pool-based elite athletes. *Med Sci Sports Exerc*. 2012;44(8):1433–9. 22297809 10.1249/MSS.0b013e31824c823c

[62] Helenius IJ Rytilä P Metso T Haahtela T Venge P Tikkanen HO. Respiratory symptoms, bronchial responsiveness, and cellular characteristics of induced sputum in elite swimmers. *Allergy*. 1998;53(4):346–52. 9574875 10.1111/j.1398-9995.1998.tb03904.x

[63] Morgado JP Monteiro CP Teles J, . Immune cell changes in response to a swimming training session during a 24-h recovery period. *Appl Physiol Nutr Metab*. 2016;41(5):476–83. 27028294 10.1139/apnm-2015-0488

